# New Insights Into Upper Messinian Microbial Carbonates: A Dendrolite‐Thrombolite Build‐Up From the Salento Peninsula, Central Mediterranean

**DOI:** 10.1111/gbi.70023

**Published:** 2025-05-25

**Authors:** Alessandro Vescogni, Francesco Colombo, Adriano Guido

**Affiliations:** ^1^ Dipartimento di Scienze Chimiche e Geologiche University of Modena and Reggio Emilia Modena Italy; ^2^ Dipartimento di Biologia, Ecologia e Scienze Della Terra University of Calabria Arcavacata di Rende Italy

**Keywords:** central Mediterranean, dendrolite, late Messinian, microbialite, thrombolite

## Abstract

Upper Messinian carbonates recently recorded in the Salento Peninsula (southern Italy, central Mediterranean) contain microbial facies, including textures never previously described in the Late Miocene of the Mediterranean. This study focuses on the geometry and internal fabrics of a 3 × 28 m build‐up of coalescent dendrolite and thrombolite, to examine its formation and the possible microbes involved, and to reconstruct its growth dynamics and related palaeoenvironmental conditions. Salento dendrolites have centimetric dendritic growth forms with a microlaminated, originally aragonitic, microstructure. The thrombolites, in contrast, are characterized by larger mesoclots with arborescent, anastomose growth patterns and a distinctive microfabric of small, originally calcitic, spheroids with a sparry nucleus surrounded by acicular crystals. Bio‐geochemical analyses (UV epifluorescence, micro‐Raman spectroscopy and SEM‐EDS) reveal the presence of organic matter intimately associated with both dendrolite and thrombolite textures, supporting a biotic origin. The sedimentary context and microfabrics suggest that cyanobacteria may have played a major role in the formation of these structures, together with heterotrophic microbes, mainly sulfate‐reducing bacteria, in the dendrolite. Build‐up geometries, stratigraphic setting, and analysis of the associated sediment suggest that the dendrolite‐thrombolite framework developed in a small, shallow‐water lagoon, under moderate to high energy, variable salinity, and possibly high sedimentation rate. Salento dendrolite‐thrombolite build‐up appears to be the only known example of large microbial bioconstruction made by microlaminated dendrolites.

## 1 Introduction

During the Late Miocene, the Mediterranean basin underwent profound environmental and biotic changes related to the progressive closure of the connection with the Atlantic Ocean, culminating in the late Messinian so‐called “salinity crisis” and deposition of a thick evaporite succession (Roveri et al. 2014, and references therein). This important series of events had a major impact on carbonate platform development, starting with the demise of upper Tortonian/lower Messinian coral reefs, which in many areas were replaced by microbial carbonates just after (Franseen et al. 1998; Bourillot et al. 2009) or in conjunction with the evaporite precipitation (Cornèe et al. 2006; Roveri et al. 2020). These microbialites have been studied mainly in the western Mediterranean, in extensive outcrops located in southern Spain and north Africa (Esteban 1979; Roep et al. 1979, 1998; Riding et al. 1991; Martin et al. 1993; Braga et al. 1995; Dabrio and Polo 1995; Calvet et al. 1996; Feldmann and McKenzie 1997; Krijgsman et al. 2001; Bourillot et al. 2020). These sites have provided important information regarding the evolution of microbialite types and fabrics, underscoring the importance of the Mediterranean late Messinian as a “laboratory” for understanding microbial carbonate development. From southern Spain, for example, some of the best documented examples of agglutinated stromatolites and thrombolites in the geological record have been reported (Riding et al. 1991; Martin et al. 1993; Braga et al. 1995). Agglutinated textures are locally well‐represented in present‐day microbialite assemblages but are practically unknown prior to the late Messinian (Riding 2000) and have been linked to a progressive increase over time of not only cyanobacteria but also micro‐algae producing extracellular polymeric substances (EPS), thereby enhancing the trapping ability of the microbial communities (Awramik and Riding 1988).

**FIGURE 1:**
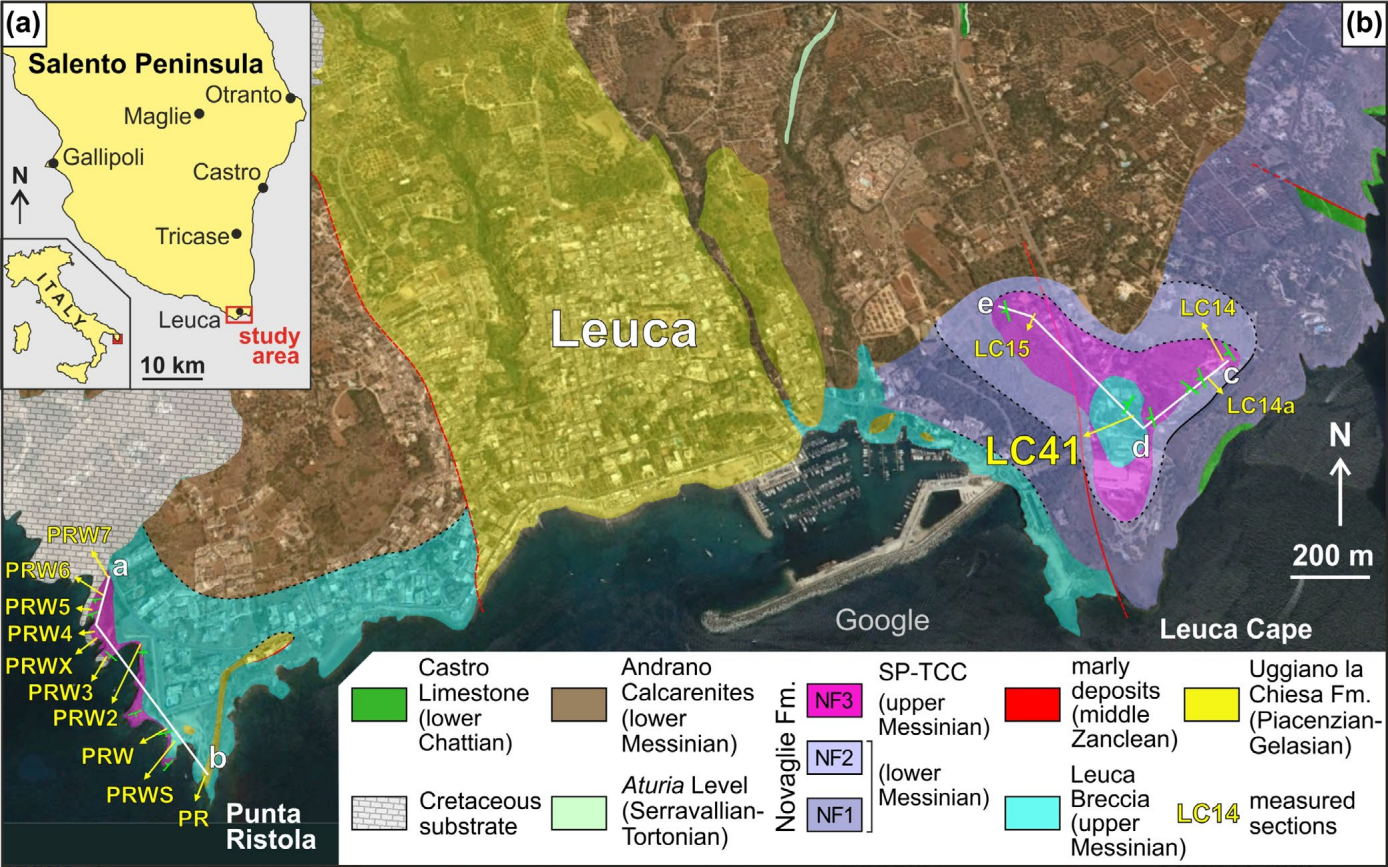
(a) Schematic map of the southern Salento Peninsula with the location of the study area. (b) Geological map of the study area showing the position of the main sections of the SP‐TCC and the trace of the three transects depicting the SP‐TCC architecture (a‐b, c‐d, d‐e) reported in Figure 3 (modified from Vescogni et al. 2022).

Upper Messinian microbial carbonates have recently been described in the Salento Peninsula (central Mediterranean, south‐eastern Italy, Figure 1a), with the identification of a diversified assemblage that includes several microbial facies. A preliminary characterization of these microbialites has provided an account of their stratigraphic architecture, palaeoenvironmental setting, and age determination (Vescogni et al. 2022). In particular, the dating was carried out using ^87^Sr/^87^Sr isotopes analyses that provided an age between 5.97 and 5.60 Ma. A remarkable outcome of this study is the discovery of microbial fabrics very different from those so far known from the late Messinian of the Mediterranean. This includes a large build‐up formed by the coexistence of dendrolites and thrombolites, the former with centimetric clusters of dendritic growth‐forms called dendroids (*sensu* Shapiro and Wilmeth 2020) with microlaminated microfabric and the latter with larger, branched mesoclots (*sensu* Kennard and James 1986) made of sub‐millimetric spheroidal microstructures. Although Vescogni et al. (2022) interpreted these textures as microbial, their precise nature has not been thoroughly investigated and remains somewhat ambiguous.

The aim of the present paper is to provide a detailed account of this build‐up: to describe its geometries and internal fabrics, to test the biogenicity of these structures, and to investigate the type and role of microbial associations possibly involved in their development. At the same time, the paleoenvironmental conditions and the growth phases leading to the formation of the Salento dendrolite‐thrombolite build‐up (DTB) are reconstructed. The study is based on a multidisciplinary approach which includes: (a) detailed description of the fabrics at macro‐, meso‐, and microscale; and (b) bio‐geochemical characterization of the microstructures through UV epifluorescence, micro‐Raman spectroscopy, SEM‐EDS, and XRPD analyses.

## 2 Geological Setting

**FIGURE 2:**
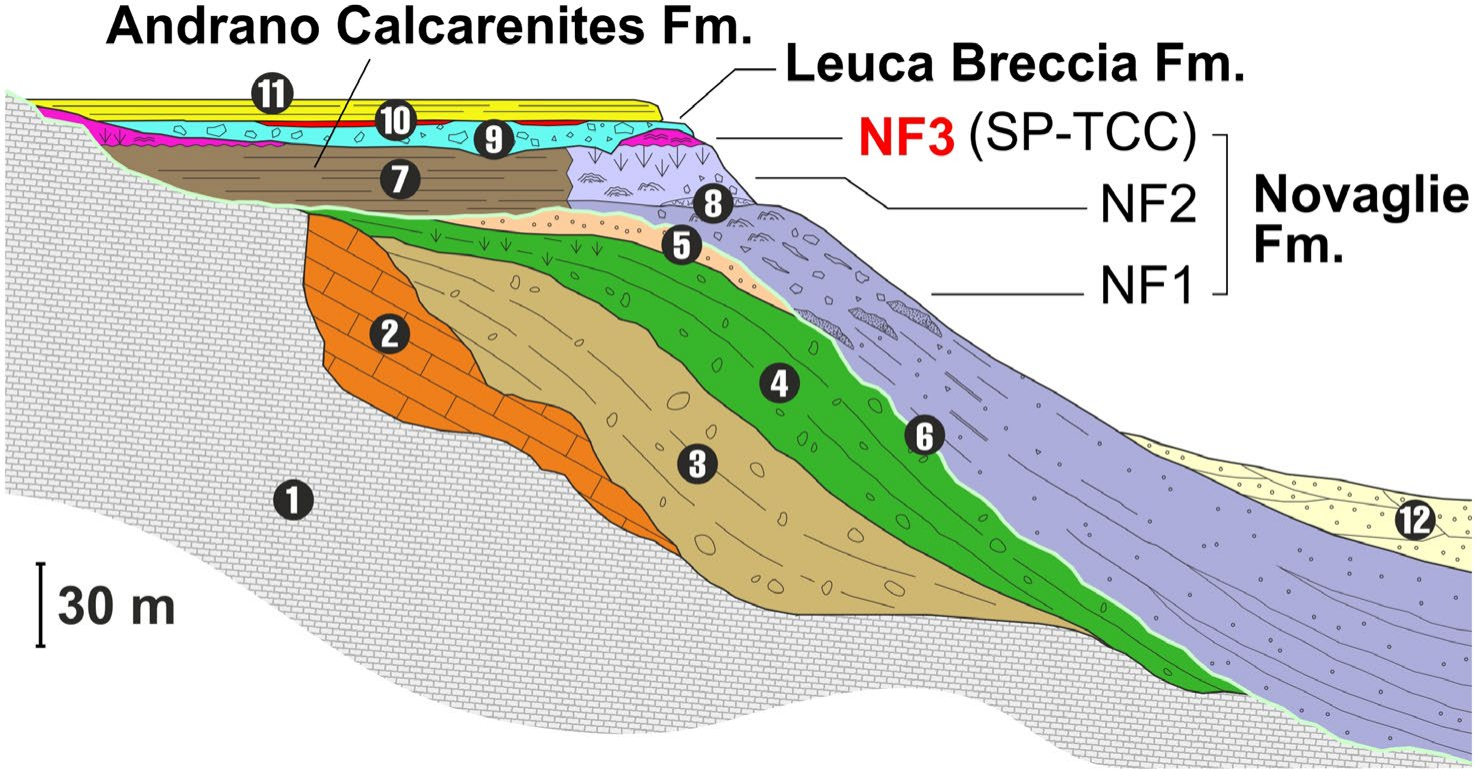
Stratigraphic setting of the eastern Salento Peninsula: 1‐Upper Cretaceous substrate, 2‐Torre Tiggiano Limestone (Lutetian/Bartonian), 3‐Torre Specchialaguardia Limestone (Priabonian), 4‐Castro Limestone (middle‐upper Chattian), 5‐Porto Badisco Calcarenite (uppermost Chattian), 6‐*Aturia* level (Serravallian/Tortonian), 7‐Andrano Calcarenites (lower Messinian), 8‐Novaglie Formation (lower/upper Messinian) which is separated into three depositional sequences NF1, 2, 3, the last one also indicated as Salento Peninsula Terminal Carbonate Complex (SP‐TCC), 9‐Leuca Breccia (upper Messinian), 10‐Trubi Fm. (middle Zanclean), 11‐Uggiano la Chiesa Fm. (Piacenzian/Gelasian), 12‐Salento and Gravina Calcarenites (Lower Pleistocene) (modified from Bosellini et al. 1999).

The Salento Peninsula constitutes the southern portion of the Apulia Platform, a major structural element at the northern boundary of the African plate. This area hosted the deposition of shallow water carbonates since the Late Triassic, and from the Late Cretaceous, further shallowing led to the formation of an isolated, partially emerged platform. In this setting, a series of relatively thin carbonate sequences developed, removed from significant terrigenous input and concentrated in the distal part of the plateau (Figure 2). These sequences mainly include clinostratified bioclastic successions and coral reef systems, Campanian/Maastrichtian to Lower Pleistocene in age, that crop out along the south‐eastern Salento coast from Otranto to the Leuca Cape (Figure 1a). A comprehensive overview of Salento stratigraphic architecture can be found in Bosellini et al. (1999), Ricchetti and Ciaranfi (2013), and Milli et al. (2024).

In the study area, the carbonate succession developed on Cretaceous basement (Figures 1b and 2) and includes coral reef slope deposits of the Castro Limestone Formation (lower Chattian; Bosellini et al. 2021 and references therein) and a thin phosphatic hardground named the “*Aturia* level” (Serravallian/Tortonian; Vescogni et al. 2018 and references therein). These lithologies are in turn followed by a series of shallow‐water Messinian sediments that belong to different formations, related to distinct environmental conditions and depositional phases, which can be summarized as follows. The Novaglie Formation was deposited along the external borders of the plateau and is made of the superimposition of three sequences (NF1/2/3) separated by erosional surfaces (Figures 1b and 2; Bosellini et al. 2001, 2002; Bossio et al. 2002; Bosellini 2006; Brachert et al. 2007; Vescogni et al. 2008, 2011; Braga et al. 2009). The two lower units are typical monogeneric *Porites* coral reefs, lower Messinian in age and respectively 120 and 20 m in thickness. Toward the inner part of the platform, these two bioconstructions pass into the Andrano Calcarenites Formation, represented by a wide belt of back‐reef bioclastic sediments. The following NF3 sequence overlies the last *Porites* reef (NF2) and consists of up to 10 m of oolitic calcirudites/calcarenites, associated with microbialites, scattered *Porites* colonizations, and small vermetid/serpulid bioherms. The NF3 unit, which contains the build‐up considered in this study, has been recently dated as late Messinian and compared to the western Mediterranean Terminal Carbonate Complex (see discussion in Vescogni et al. 2022); for this reason, the NF3 has been named the Salento Peninsula Terminal Carbonate Complex (SP‐TCC). The SP‐TCC is overlain by the Leuca Breccia Formation, an up to 12‐m thick breccia composed of clasts derived from the underlying Novaglie Formation and considered to be the result of sea‐level fall associated with the late Messinian salinity crisis (Bosellini et al. 1999; Vescogni et al. 2022; Milli et al. 2024). The Leuca Breccia Formation is followed by some dm‐thick, discontinuous deposits of middle Zanclean marls and by the Upper Piacentian‐Gelasian Uggiano la Chiesa Formation, consisting of tens of meters of calcarenite with a basal breccia (Bosellini et al. 1999).

## 3 SP‐TCC Stratigraphic Setting and Carbonate Facies

**FIGURE 3:**
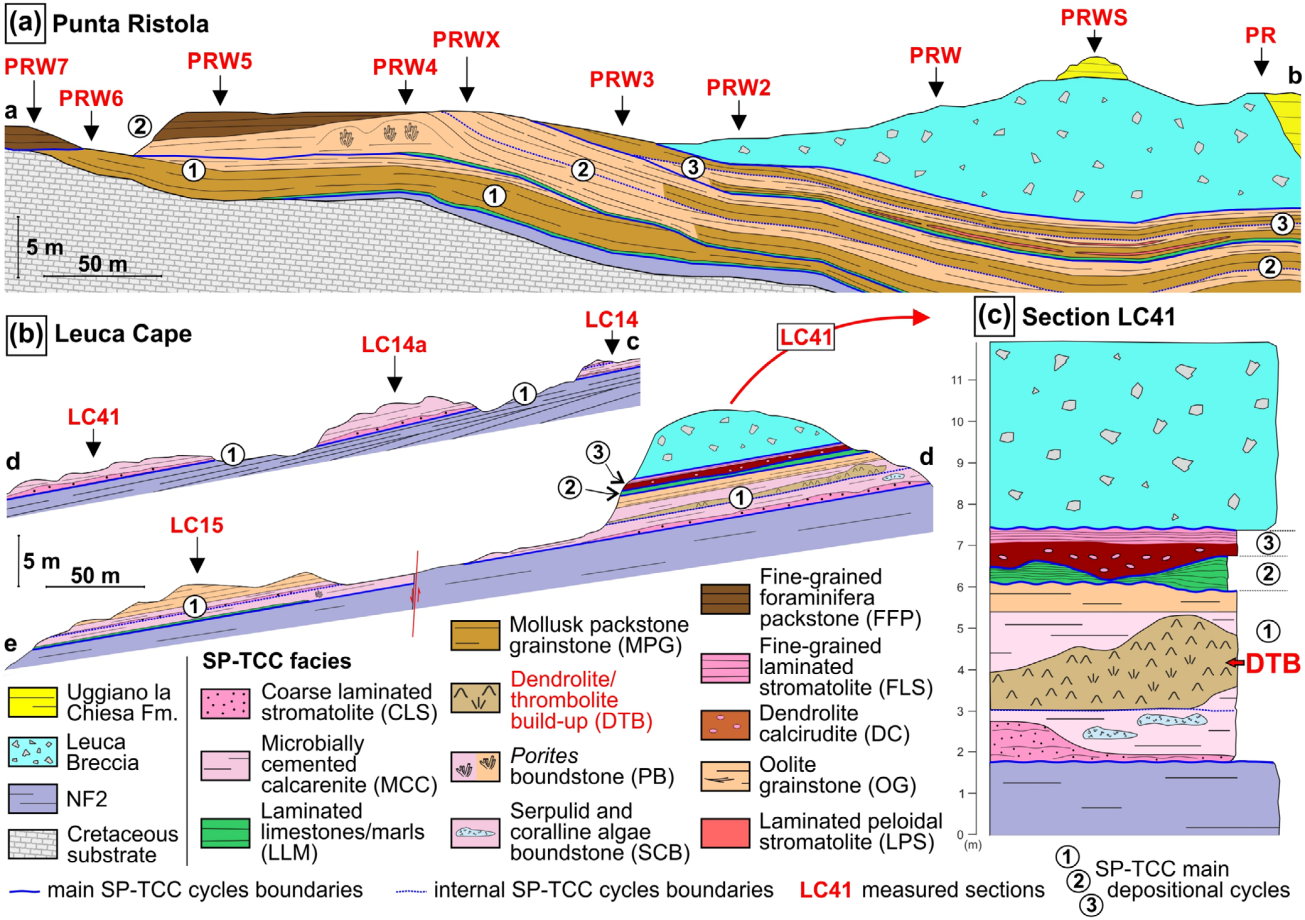
SP‐TCC stratigraphic settings: (a) Punta Ristola (transect a‐b), (b) Leuca Cape (transects c‐d, d‐e). (c) LC41 section showing the stratigraphic position of the dendrolite‐thrombolite build‐up (modified from Vescogni et al. 2022).

The SP‐TCC crops out at both sides of the Leuca Gulf (Table 1; Vescogni et al. 2022). Three stratigraphic transects (Figures 1b and 3) display the succession of three small‐scale depositional sequences, separated by erosion surfaces related to high‐frequency eustatic oscillations. In addition, the occurrence of higher frequency cycles is sometimes indicated by the alternate superimposition of some of the SP‐TCC facies (Figure 3).

**TABLE 1:** Summary of sedimentary and palaeontological features of SP‐TCC facies and palaeoenvironmental conditions (modified from Vescogni et al. 2022).

Facies	Geometries and sedimentary features	Carbonate grains	Palaeoenvironmental conditions
LLM Laminated limestones/marls	Superimposition of centimetric fine‐grained laminated limestone (mainly mudstone) and marls layers; maximum overall thickness of 60 cm	Rare within the limestones: gastropods, *Elphidium*, *Ammonia*, muddy intraclasts, echinoderms, ostracods, bivalves, coralline and dasycladacean algae, serpulids, brachiopods, pellets, and oolites. Almost absent within the marls: *Ammonia*, *Elphidium*	Shallow‐water, euphotic conditions; low to moderate hydrodynamic setting; salinity from normal marine (limestones) to brackish (marls)
MPG Mollusk packstone‐grainstone	Stratified packstone‐grainstone layers, up to 3.8 m of overall thickness	Gastropods, small intraclasts, coralline algae, bivalves, pellets, miliolids, *Elphidium, Ammonia* and other small benthic foraminifera, echinoderms, encrusting foraminifera, brachiopods, bryozoans, serpulids, oolites, dasycladacean algae, *Porites* fragments	Shallow‐water, euphotic conditions; moderate to high‐energy hydrodynamic setting; normal marine salinity
OG Oolite grainstone	Stratified grainstone deposits with planar and cross bedding, up to 5 m of overall thickness	Oolites with nuclei made of bivalves, pellets, intraclasts, gastropods, miliolids, coralline algae, *Elphidium*, *Ammonia*, echinoderms, dasycladacean algae, and serpulids	Shallow‐water, euphotic conditions; high‐energy hydrodynamic setting; normal marine salinity
FFP Fine‐grained foraminifera packstone	Stratified packstone deposits, up to 2 m thick	Gastropods, pellets, miliolids, *Elphidium*, *Ammonia* and other small rotaliids, coralline algae, echinoderms, bivalves, muddy intraclasts, small oolites, ostracods, brachiopods, dasycladacean algae, bryozoans, and serpulids	Shallow‐water, euphotic conditions; moderate hydrodynamic setting; normal marine salinity
PB *Porites* boundstone	Coral boundstones up to 1.8 m thick and 40 m wide	*Porites* and *Siderastrea* colonies, oolites, pellets, coralline algae, gastropods, bivalves, echinoderms, miliolids, brachiopods, serpulids, and small intraclasts	Moderate to high‐energy hydrodynamic setting and normal marine salinity
LPS Laminated peloidal stromatolite	Laminated sub‐horizontal peloidal stromatolitic crusts up to 10 cm of overall thickness	Oolites, pellets, gastropods, bivalves, and small benthic foraminifera	Moderate hydrodynamic conditions, possible fluctuations in salinity and/or oxygenation
CLS Coarse laminated stromatolite	Coarse stromatolites forming some dm thick sub‐horizontal layers and up to 1 m thick domal structures, both with an inner laminated arrangement	Gastropods, bivalves, coralline red algae, pellets, oolites, echinoderms, intraclasts, serpulids, miliolids, *Halimeda*, *Porites* fragments, brachiopods, bryozoans, *Elphidium*, *Cibicides*, *Ammonia* and other small rotaliids, encrusting foraminifera, dasycladacean algae	Shallow‐water, euphotic conditions; moderate to high‐energy hydrodynamic setting; normal marine salinity
MCC Microbially cemented calcarenit	Massive to weakly stratified coarse grainstones stabilized by microbial peloids, up to 3 m in overall thickness	Gastropods, bivalves, coralline red algae, pellets, oolites, echinoderms, intraclasts, serpulids, miliolids, *Halimeda*, *Porites* fragments, brachiopods, bryozoans, *Elphidium*, *Cibicides*, *Ammonia* and other small rotaliids, encrusting foraminifera, dasycladacean algae	Shallow‐water, euphotic conditions; high‐energy hydrodynamic setting; normal marine salinity
SCB Serpulid and coralline algae boundstone	Massive, lenticular boundstones up to 70 cm thick and 200 cm in width	Serpulids, coralline red algae, vermetids, pellets, miliolids, *Elphidium*	Moderate to high‐energy hydrodynamic setting; normal marine salinity
DTB Dendrolite/thrombolite build‐up	Massive dendrolite/thrombolite build‐up 80 to 300 cm thick	Oolites, pellets, peloids, molluscs, small benthic foraminifera (miliolids, *Elphidium* and *Ammonia*)	Shallow‐water, euphotic conditions; moderate to high hydrodynamic setting, variable salinity
DC Dendrolite calcirudite	Massive dendrolite rudstone deposits with the packstone matrix, up to 85 cm of overall thickness	Bivalves, echinoderms, miliolids, and other rotaliids, dendrolites fragments, gastropods, *Elphidium*, ostracods, serpulids, and bryozoans	Shallow‐water, euphotic conditions; moderate to high hydrodynamic setting; normal marine salinity
FLS Fine‐grained laminated stromatolite	Superimposition of fine‐grained stromatolites arranged in centimetric laminated layers with sparse bioclastic horizons; overall thickness up to 35 cm	Pellets, gastropods, bivalves, miliolids and other small benthic foraminifera, echinoderms	Shallow‐water, euphotic conditions; moderate hydrodynamic setting; normal marine salinity

Along the western side of the gulf, near Punta Ristola, the SP‐TCC succession is up to 10 m thick and rests on NF2 bioclastic sediments or directly overlies the Cretaceous basement (Figure 3a). Each of the three main cycles starts with a thin layer of laminated limestone/marls (LLM), followed by clinostratified deposits made of molluscan packstone‐grainstone (MPG), oolite grainstone (OG), and fine‐grained foraminiferal packstone (FFP). Within these bioclastic deposits, a patch of *Porites* boundstone (PB) and thin horizons of laminated peloidal stromatolites (LPS) can also be found. A sharp erosional surface marks the transition to the overlying Leuca Breccia Formation.

At the opposite side of the gulf, in the Leuca Cape area, the SP‐TCC is up to 6 m thick and developed over *Porites* bioconstructions and slope deposits of the NF2 (Figure 3b). Here it shows a complex succession that includes eight different facies. This is mainly visible along the LC41 stratigraphic section (Figure 3c) where the basal cycle starts with crusts and small mounds of coarsely laminated stromatolite (CLS). This facies interfingers with and is overlain by microbially cemented calcarenites (MCC) and discontinuous lenses of serpulid and coralline algae boundstones (SCB). The succession continues with the massive dendrolite‐thrombolite build‐up (DTB) described in this paper, which is surrounded and covered by MCC. Stratified deposits of OG overlie the latter facies. The second SP‐TCC sequence shows a reduced thickness and includes a single layer of LLM. A very irregular surface marks the base of the final cycle, made of a basal deposit of dendrolite calcirudite (DC) capped by some decimeters of fine‐grained laminated stromatolite (FLS). As in Punta Ristola, a clear unconformity marks the boundary between the top of the SP‐TCC and the Leuca Breccia Fm.

## 4 Materials and Methods

### 4.1 Dendrolite‐Thrombolite Build‐Up Stratigraphy, Geometries, and Fabrics

**FIGURE 4:**
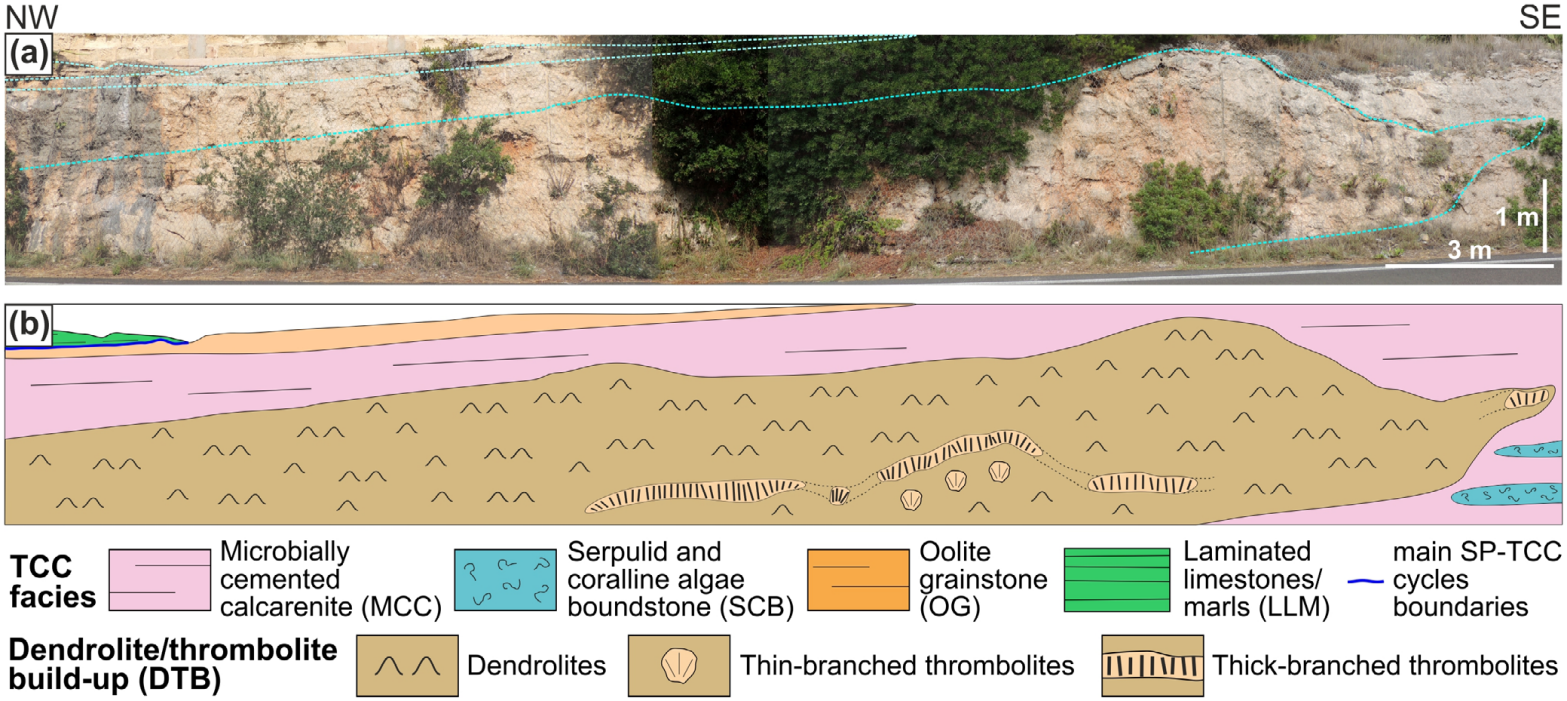
(a) Dendrolite‐thrombolite build‐up along the LC41 section. (b) Schematic representation of the DTB showing the distribution of the dendrolite and thrombolite facies, and the stratigraphic relationships with the adjacent SP‐TCC facies.

The DTB crops out along the roadcut just north of Leuca Cape (section LC41‐39°47′51.49″N/18°22′10.89″E) (Figures 1b and 3c). Since this is a vertical section (Figure 4), it limits perception of the three‐dimensional development of the build‐up. Even so, detailed observations allowed the characterization of a large portion of this structure. Dendrolites and thrombolites have been described at various scales, distinguishing their macrofabrics (meters to decimeters), mesofabrics (centimeters to millimeters) and microfabrics (millimeters to tens of millimeters). Macrofabric scale data have been acquired in the field: DTB size and shape, arrangements of the dendrolite and thrombolite textures and their relations with the surrounding facies. Mesofabrics have been investigated both in the field and by analysis of decimetric hand samples in terms of growth forms, size, shape, orientation and their relations with the associated sediment. DTB microfabrics have been characterized by optical microscope (Leitz Orthoplan) analysis of 32 thin sections (6 × 4.5 cm) at differing magnifications (16×, 25×, 40×, 63×, and 100×), with description of dendrolite and thrombolite micro‐assemblages and internal fabric. The same samples have been used to identify the associated sediment composition and texture, using Dunham's (1962) classification.

### 4.2 UV‐Epifluorescence

UV‐epifluorescence allows for identification of the presence and distribution of organic compounds within mineral structures and is useful in discriminating between mineral precipitation of biotic origin and that related to abiotic depositional processes (e.g., Neuweiler and Reitner 1995; Russo et al. 1997; Guido et al. 2022, 2024; Cipriani et al. 2024), especially in cases where the general aspect of these products appears similar in reflected or transmitted light.

Small slabs and uncovered thin sections used for microfabric analyses were also utilized to investigate the occurrence of organically activated luminescence. Incident light, emitted by a Hg high‐pressure vapor bulb, attached to a Zeiss Axioplan Imaging II microscope, with high‐performance wide bandpass filters, was used as a parameter of fluorescence intensity (bandpass filter 436/10 nm/long‐pass filter 470 nm, no. 488006, for the green light; and bandpass filter 450–490 nm/long‐pass filter 515 nm, no. 488009, for the yellow light).

### 4.3 Micro‐Raman Spectroscopy

Coupled with UV‐epifluorescence, Raman spectroscopy has been used to recognize the presence of organic compounds and distinguish autochthonous carbonates, mineralized in situ through putative microbial mediation, from the allochthonous components, transported from external sources (Guido et al. 2022). Micro‐Raman analyses were also performed on polished slabs and thin sections, with a focus on dendrolite and thrombolite microfabrics and on detrital sediment and secondary sparry calcite. We used a Thermo Fisher DXR Raman microscope (Waltham, MA, USA), equipped with OMNICxi Raman Imaging software 1.0, an objective of 50×, a grating of 900 ln/mm (full width at half maximum, FWHM), and an electron multiplying charge‐coupled device (EMCCD). The 532 nm line (solid state laser) was used at an incident power output ranging from 1.8 to 7 mW. The spatial resolution of the laser beam was about 3–5 μm. The acquisition time of the spectra varied from 5 to 40 s. Data were collected in the 50–3360 cm^−1^ range to capture the first order and second order Raman bands. The measurements were collected on randomly oriented grains, with a fixed orientation of the polarized laser beam.

### 4.4 Scanning Electron Microscopy (SEM)–Energy‐Dispersive X‐Ray Spectroscopy (EDS)

SEM and EDS were performed to characterize the morphology and size of the carbonate crystals within dendrolite and thrombolite microfabrics and to investigate their mineralogical and chemical composition. For this purpose, some selected samples were carbon‐coated and analyzed by an Ultra High Resolution SEM (UHR‐SEM)—ZEISS CrossBeam 350 under the following conditions: resolution 123 eV, high voltage 10 keV, probe current 100 pA, and working distance 11 mm. Crystal compositions were detected using the following conditions: voltage 15 keV, probe current 10 nA, working distance 12 mm, take‐off angle 40°, live time 30 s.

### 4.5 X‐Ray Powder Diffraction (XRPD) Analyses

Powdered samples were analyzed by XRPD to determine and quantify the main mineralogical phases of the dendrolites and thrombolites. Briefly, about 100 mg of bulk sample was ground with an agate pestle and mortar to produce a homogeneous powder with an average grain size of a few microns. Each powdered sample was gently mounted in the central hole of a cylindrical zero background sample holder, made of oriented Si monocrystalline wafer. The XRPD patterns were obtained using a 3rd generation θ–θ Bragg–Brentano Empyrean diffractometer (Malvern PANanalytical) equipped with a multipurpose sample holder, PIXcel3D detector, MultiCore Optics (iCore and dCore) and exploiting a CuKα (Ni filtered) radiation produced at 40 kV and 40 mA. Data were collected in the 3°–80°2θ range using a step scan of 0.0131303°2θ and a counting rate of 74 s/step. Beam masks at 14 mm and 6 mm and a 0.03° Soller slit were used on the incident beam. A 0.04° Soller slit was used on the diffracted beam. 1/2° divergence slits and an antiscatter slit were used. Quantitative phase analyses (QPA) of all the considered samples were obtained following Rietveld—RIR refinement (Gualtieri 2000; Gualtieri et al. 2019) performed using GSAS software with EXPGUI interface (Larson and Dreele 2000).

## 5 Results

### 5.1 DTB Stratigraphy, Geometries, and Fabrics

The DTB has a massive appearance and mound‐like shape and crops out for a total visible length of about 28 m and a maximum thickness of about 3 m (Figure 4). The build‐up is completely surrounded by grainstone deposits of MCC facies, with contacts marked by a gradual but rapid decrease in the amount of dendrolitic and thrombolitic structures. This lack of sharp boundaries is further accentuated by the similarity between DTB inner sediment and the surrounding MCC, since in the field they both appear as yellowish, grain‐supported calcarenites. Dendrolites form the largest part of the build‐up, while thrombolites are less abundant and are mainly concentrated along a few dm thick horizons in the lower half of the DTB (Figure 4).

#### 5.1.1 Dendrolites

**FIGURE 5:**
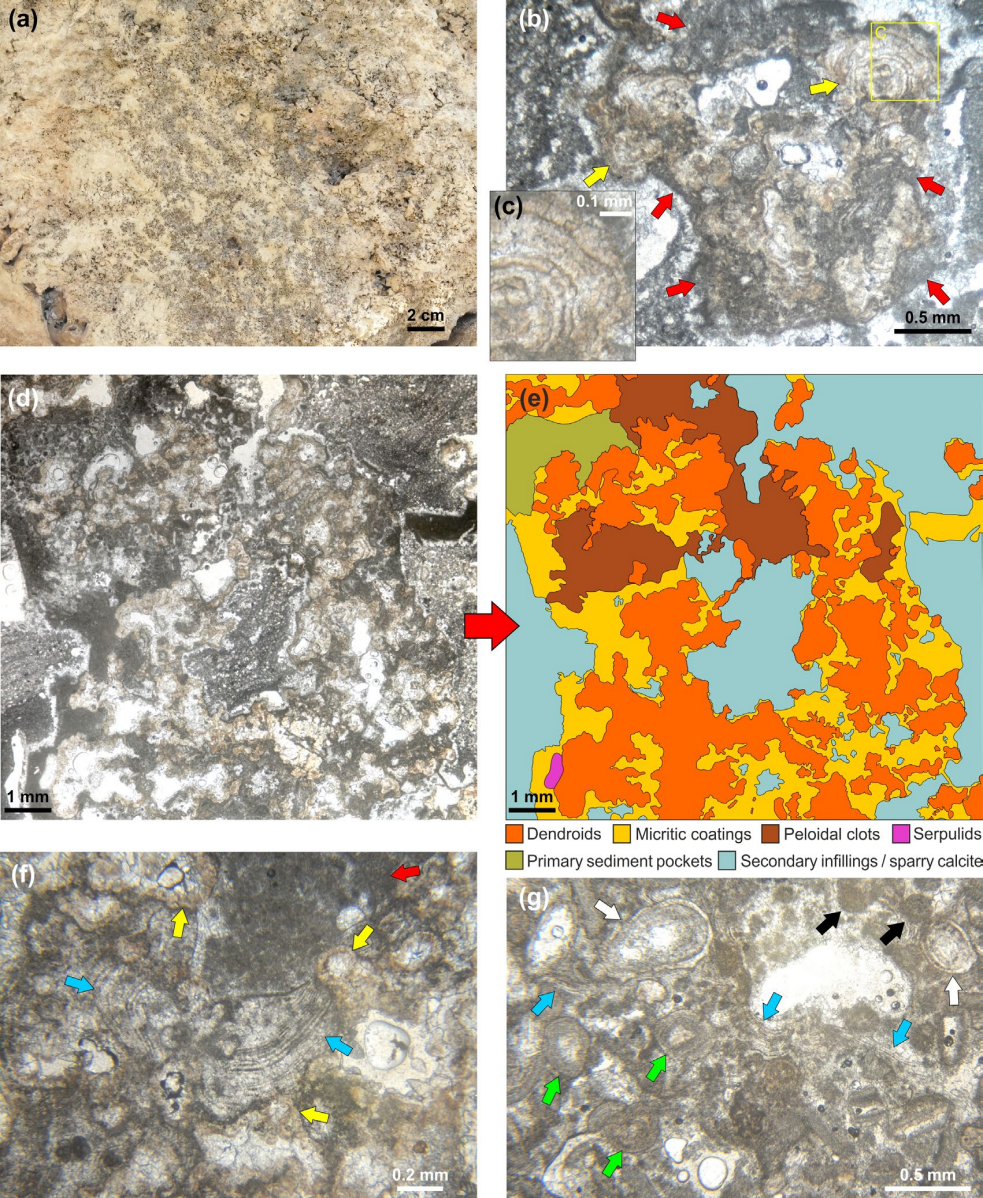
(a) Outcrop close‐up of dendroid clusters (grey/brown) within sediment (light yellow). (b) Dendroid in thin section showing its shape and internal structure; red arrows indicate micritic coatings on its surface. Note the development of the laminae following the branch profile (yellow arrows) and enveloping the dendroid branch. (c) Close‐up of a dendroid branch with alternating dark and light laminae. (d) Dendrolite framework in the thin section. (e) Simplified schematic of (d) showing type and arrangement of components forming the dendrolite framework. (f) Thin section with dendrolite dendroids (yellow arrows) coated by micritic crusts with dark and lighter laminae (blue arrows) and aphanitic/peloidal micrite (red arrow). (g) Thin section showing a grainstone‐packstone pocket amid dendroids; the most common grains are ooids (green arrows), gastropods (white arrows), and pellets (black arrows). Laminated micritic crusts (blue arrows) bind the bioclasts.

DTB dendrolites appear as an irregular superimposition of centimetric clusters of small dendroids, darker in color than the surrounding sediment (Figure 5a). They form a massive, three‐dimensional framework, in which dendroids can be closely packed or more widely spaced, leaving space for small sediment pockets. These variations in density are randomly distributed, and no alignments or other spatial arrangements were observed. In thin section, each dendroid appears as an aggregation of small branches (Figure 5b), a few mm to 1 cm in size, rarely exceeding 2 cm. The individual branches are short, generally upward‐oriented, with upward‐widening sides and marked angles of divergence that give the dendroids a moderate height/width ratio (Figure 5b). Branches of adjacent dendroids may be in close contact, but do not merge. Traces of abrasion and bioperforation are extremely rare. Dendroid microfabric is fine‐laminated; each branch is made of a distinct alternation of very thin dark laminae (few μm in thickness) and slightly thicker, lighter colored ones (up to 30 μm thick) (Figure 5c). These laminae develop roughly parallel to the branch margin, and during their growth, a new generation of laminae partially or completely envelops the dendroid branch (Figure 5b). Occurrence of sediment particles within the microfabric is extremely rare.

Dendroids are subsequently enveloped by crusts of aphanitic micrite or peloidal clots, which coat the branches and partially fill the cavities among them (Figure 5b,d,e). In addition, micritic crusts may often exhibit alternations of thin dark and light laminae, resembling the inner pattern of the dendroids microfabric (Figure 5f). Although these coatings rarely exceed 1 mm in thickness, they can be abundant (Figure 5d,e), thus contributing to the strengthening of the dendrolitic framework. Very rare serpulid crusts have also been observed.

Among the framework, fine‐grained grainstone‐packstone accumulated (Figure 5g) with abundant ooids, pellets, peloids, fragments of gastropods and bivalves, together with sparse small benthic foraminifers (mainly miliolids, *Elphidium* and *Ammonia*). Sometimes the laminated micritic crusts on the dendroids extend laterally into these sediment pockets, encrusting and binding the bioclastic fraction (Figure 5g). Numerous small, primary cavities are scattered among the dendrolitic framework and the related sediment, each bordered by a rim of dog tooth calcite cements and subsequently filled by a secondary fine‐grained, laminated sediment or by sparry calcite (Figure 5d,e).

#### 5.1.2 Thrombolites

Two types of thrombolite have been identified within the DTB: thin‐branched (a) and thick‐branched (b).

**FIGURE 6:**
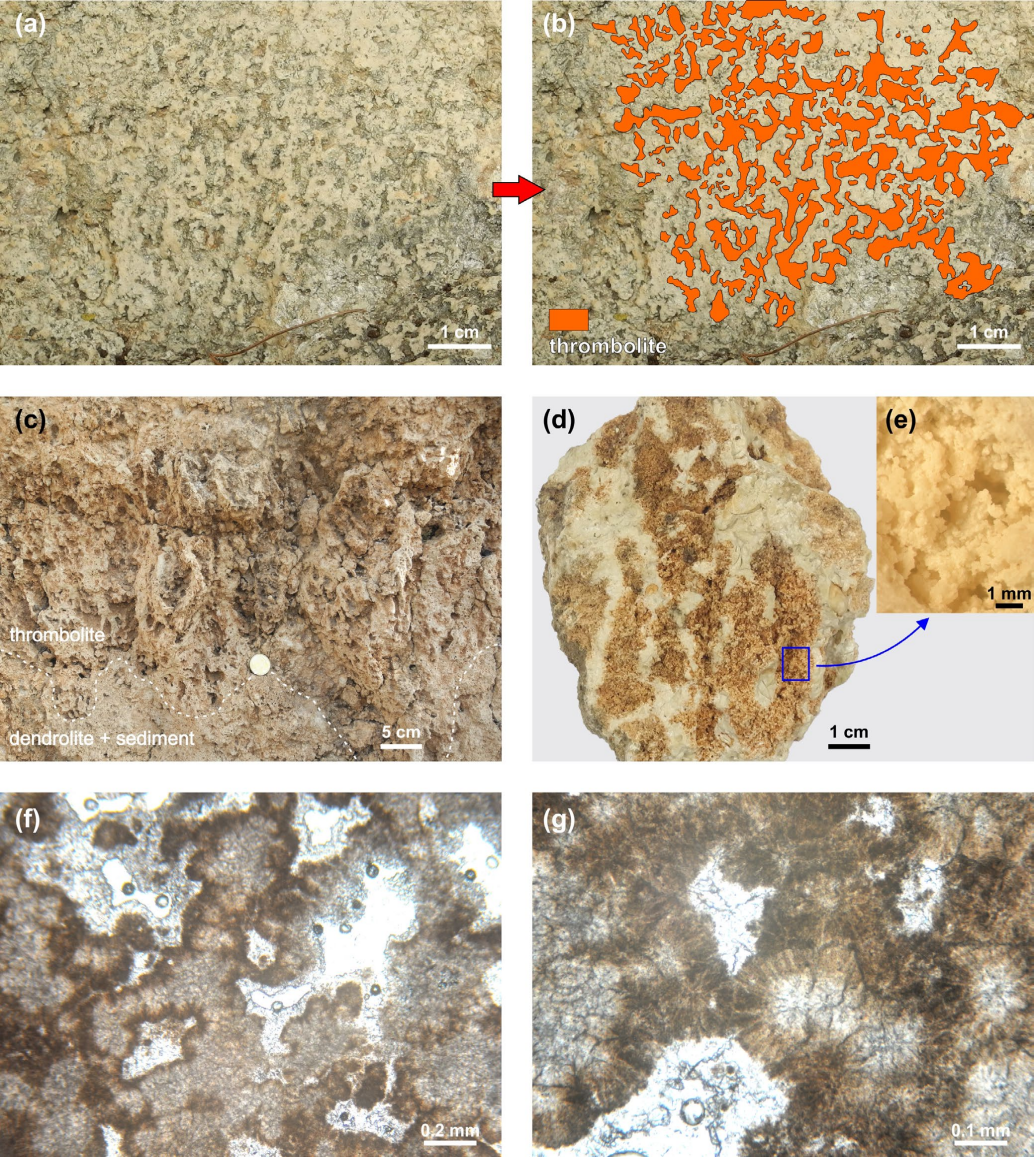
(a) Outcrop close‐up of the thin‐branched thrombolite (grey/brown) and associated sediment (light yellow). (b) Elaboration of (a) highlighting the thrombolite branched mesoclots. (c) Outcrop view of the thick‐branched thrombolite mesofabric. (d) Hand sample of thick‐branched thrombolite showing the anastomose growth pattern of the mesoclots (brown) and associated sediment (light yellow). (e) Detail of (d) showing thrombolitic spheroidal microstructure. (f) Thin section showing anastomose spheroidal thrombolitic microfabric. (g) Thin section detail of the thrombolitic spheroids with the sparry calcite inner core surrounded by acicular crystal rims.

- Thin‐branched thrombolite is relatively rare and occurs within the lower part of the build‐up, scattered among the dendrolite framework (Figure 4b). At the macrofabric scale, it appears as sub‐spherical growth forms, up to 15 cm in diameter, lacking a sharp, definite boundary with the surrounding sediment. Its presence can be detected mainly by the mesofabric organization, made of mesoclots organized in branches, few millimeters thick, having an upward‐oriented fan‐like arrangement (Figure 6a,b). During their development, the branched mesoclots frequently produce ramifications that, in turn, may converge with adjacent ones, forming an anastomose growth pattern (*sensu* Grey and Awramik 2020).
- Thick‐branched thrombolites are more abundant, and at macrofabric scale appear as large, flat bodies (up to 320 cm wide and 50 cm thick), apparently organized along a sub‐horizontal, undulated horizon that crosses the lower half of the DTB (Figure 4b). As for the thin‐branched growth forms, these lack a clear, sharp boundary with the surrounding sediment, but their outlines are more evident because of the larger size of their branched mesoclots, which are up to 1 cm in diameter and some dm in height (Figure 6c). They have a vertical growth direction with a well‐defined anastomose pattern that creates an intricate, three‐dimensional framework (Figure 6c,d).

Traces of abrasion are very rare on the surfaces of the mesoclots of both thin‐branched and thick‐branched thrombolites, and no signs of biotic crusts and bioperforation have been observed. The two thrombolite types also share similar microfabric, a clotted aggregation of small spheroidal to botryoidal structures about 0.2 mm in average diameter (Figure 6e). The interconnection of these structures creates a three‐dimensional network of agglomerations and ramifications that diverge and recombine, forming—at a microscopic scale—an anastomose pattern recalling that of the mesofabric (Figure 6f). This structure hosts a large amount of small primary cavities filled by sparry calcite. The inner portion of the spheroids has a sparry, light colored center, passing into an external rim of dark yellow acicular crystals (Figure 6f,g). No carbonate grains occur within the thrombolitic microfabric. The associated sediment, nested between the thrombolite mesoclots, is fine‐grained grainstone‐packstone similar to that described among the dendrolites.

### 5.2 UV‐Epifluorescence

#### 5.2.1 Dendrolites

**FIGURE 7:**
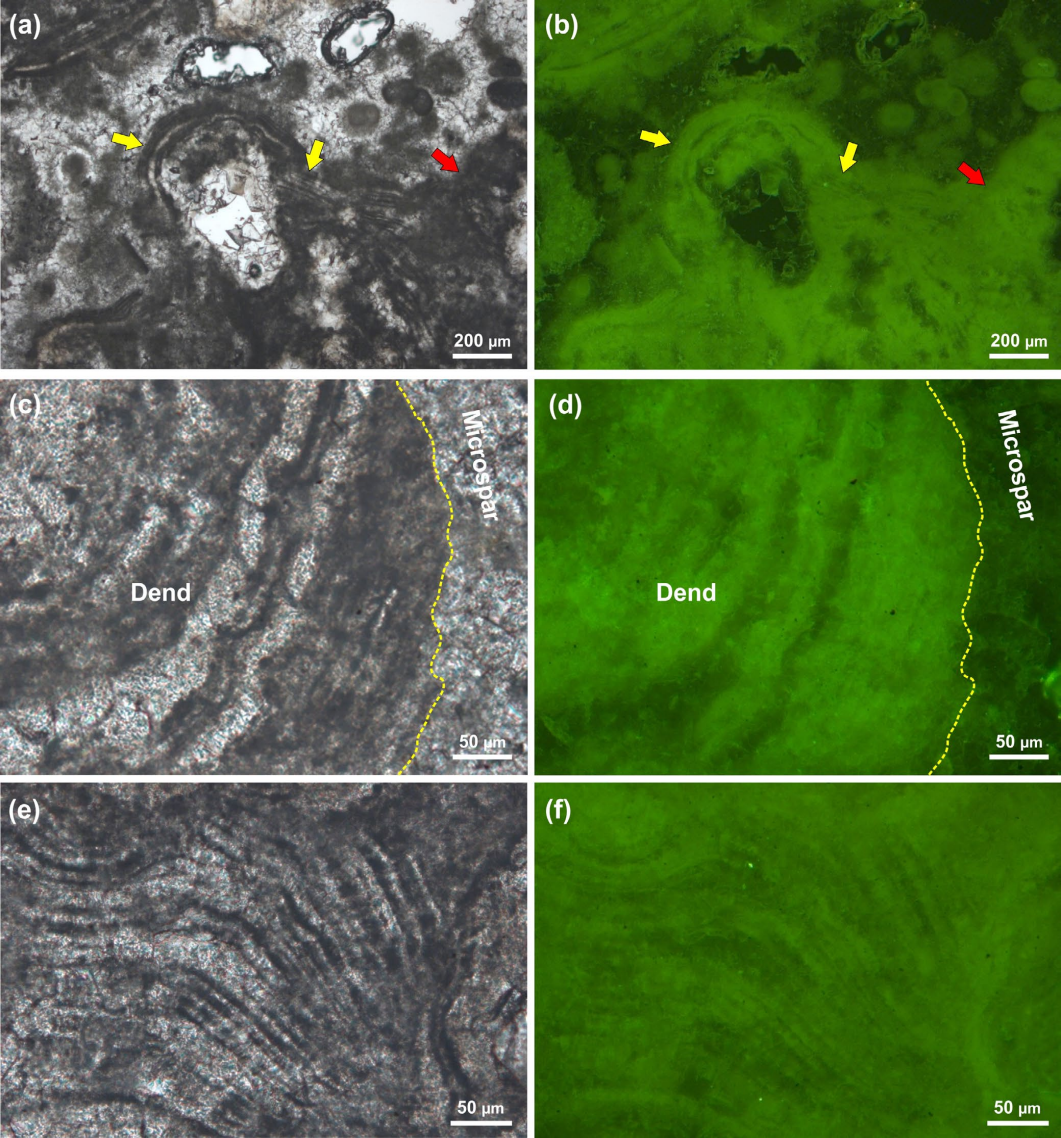
(a) Thin section of laminated dendroid branch (yellow arrows) encrusted by aphanitic/peloidal micrite (red arrow) and associated peloid microspar. (b) View of (a) under UV‐excitation, note the bright fluorescence emitted by the dendroid dark laminae (yellow arrows), micritic crust (red arrow) and peloids, which contrasts with lack of emission from the lighter laminae and microsparite. (c) Thin section view of a dendroid branch in close‐up showing alternating dark and light laminae. (d) View of (c) under UV‐excitation showing the bright epifluorescence of the dark laminae. (e) Thin section detail of laminated micritic crust with alternating dark and light laminae. (f) View of (e) under UV‐excitation showing bright epifluorescence of the dark laminae.

The fine‐laminated microfabric of the dendroids and the aphanitic/peloidal to laminated texture of the surrounding crusts are evident in reflected and transmitted light, both on plane slabs and thin sections (Figure 7a,c,e). Under UV‐excitation, the dark and lighter bands of the laminations, both in the dendroids (Figure 7b,d) and in the surrounding crusts (Figure 7f), exhibit variable epifluorescence. The dark bands are characterized by bright fluorescence in comparison to the lighter, which show no evidence of epifluorescence (Figure 7b,d,f). Due to diagenetic processes, the boundaries between the dark and lighter bands are not always well defined. The aphanitic to clotted peloidal micrite of the crusts shows similar epifluorescence under UV‐light (Figure 7b). As for the light laminae, the microsparite among the peloids and sparite filling the microcavities do not emit epifluorescence (Figure 7b,d). The emission of radiation in the visible spectrum of the dark laminae and aphanitic/peloidal micrite is linked to biomolecules strictly associated with the formation of the crystals.

#### 5.2.2 Thrombolites

**FIGURE 8:**
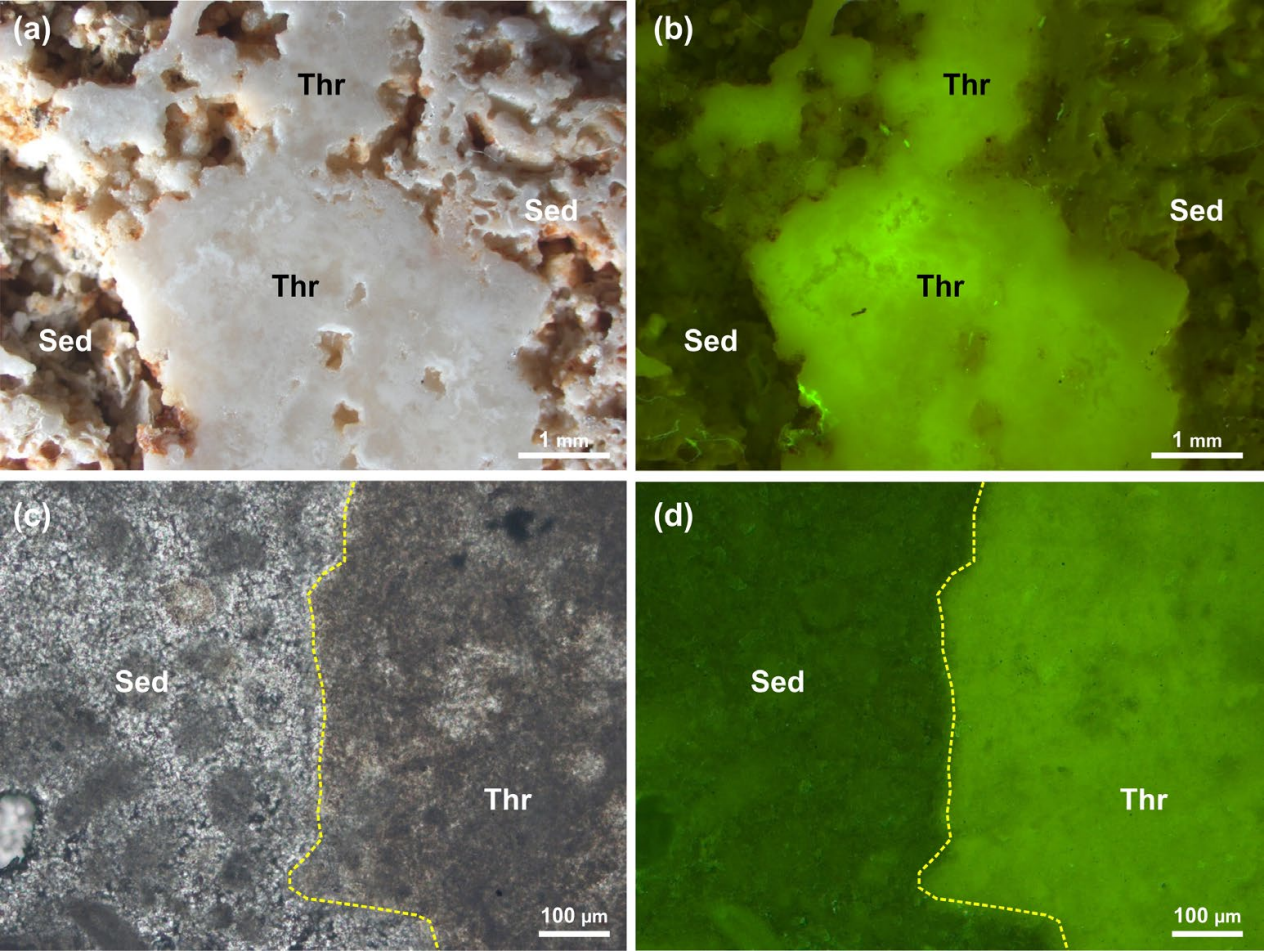
(a) Detail of a thrombolite (Thr) mesoclot in plane light with surrounding sediment (Sed). (b) View of (a) under UV‐excitation showing bright fluorescence of the thrombolite mesoclot contrasting with non‐fluorescent adjacent sediment. (c) Thin section of the boundary between thrombolite mesoclot and associated sediment. (d) View of (c) under UV‐excitation showing bright fluorescence limited to the thrombolite.

A clear distinction between the compact microcrystalline structure of the thrombolite mesoclots and the porous granular texture of the adjacent sediment is observable on small unpolished fractured fragments and plane slabs from the internal, better preserved portion of the samples (Figure 8a). The boundary between the two components is highlighted by pervasive oxidation suffered by the sediment and can be easily traced also in thin section (Figure 8c). Observations of both plane slabs and under transmitted light microscopy reveal a completely different behavior to that of thrombolite mesoclots and sediment under UV‐light. The former show a very bright fluorescence, testifying to the presence of organic matter within their microstructure, which contrasts with the non‐fluorescent, inorganic sediment (Figure 8b,d).

### 5.3 Micro‐Raman Spectroscopy

**FIGURE 9:**
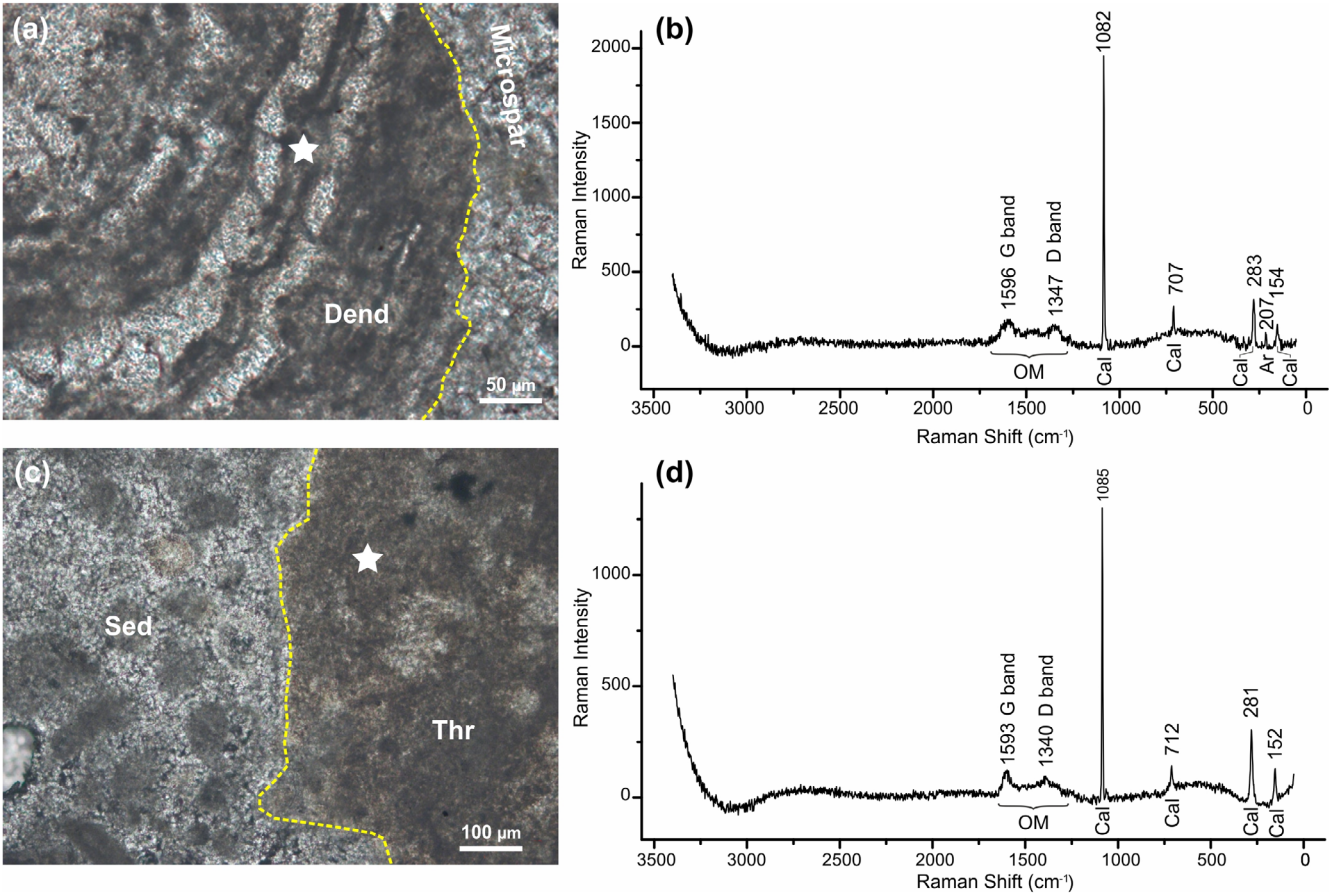
(a) Thin‐section of microlaminated dendroid branch (Dend) surrounded by microspar; star indicates the dark lamina analyzed by micro‐Raman spectroscopy. (b) Raman spectra of the dark lamina of the dendroid branch showing G‐ and D‐bands related to organic matter (OM), and four main calcite peaks (Cal); note a minor peak around 207 cm^−1^ related to the presence of aragonite crystal remains (Ar). (c) Thin‐section of a thrombolite mesoclot (Thr) and surrounding sediment (Sed); star indicates the mesoclot portion analyzed by micro‐Raman spectroscopy. (d) Raman spectra of the thrombolite mesoclot showing G‐ and D‐bands related to organic matter (OM), and four main calcite peaks (Cal).

Both dendroid laminations and thrombolite mesoclots spheroidal microstructures show Raman spectra with band positions that match the values of calcite reference bands (Figure 9). The detected peaks are located in the range between 50 and 3360 cm^−1^. The main calcite peaks correspond to the symmetric stretching (ν1) of the CO_3_ group at ~1100 cm^−1^, asymmetric stretching (ν3) at ~1450 cm,^−1^ and symmetric deformation (ν4) at ~710 cm^−1^. The lower wave numbers for calcite (~280 cm^−1^) arise from the external vibration of the CO_3_ group that involves translatory oscillations of the group. Four prominent absorption bands were recorded in the analyzed samples around 150, 280, 710, and 1085 cm^−1^ (Figure 9b,d). Dendroid laminations show a weak peak around 205 cm^−1^, conforming with characteristic aragonite spectral signals (Figure 9b). Minor shifts in the positions of the calcite bands between the analyzed samples and the spectra published in the literature may be due to the effects of natural impurities present in the sample (Buzgar and Apopei 2009; Guido et al. 2022). Characteristic bands indicating the presence of organic matter were recorded in the dark laminae of dendroids and in the external, darker portion of the thrombolite mesoclots spheroids. These components show distinctive peaks around 1595 cm^−1^, related to the presence of the G band, and around 1345 cm^−1^, related to the D band (Figure 9b,d). These peaks are characteristic of amorphous carbon (AC) and record the presence of residual organic matter. The spectra characterized by the presence of G and D bands are also marked by pronounced bumps in the region between 430 and 850 cm^−1^ and in the region between 1250 and 1650 cm^−1^. The organic G and D bands were not recorded in the spectra of non‐fluorescent sparry calcite and detrital sediments of either microfacies. In summary, the presence of G and D bands of amorphous carbon in the putative microbial textures, where high epifluorescence was also observed, confirms the presence of organic matter relics in these components.

### 5.4 XRPD and SEM‐EDS

#### 5.4.1 Dendrolites

The main mineralogical phases of the dendrolite sample were recognized via XRPD analyses. The most intense reflections are attributable to the calcite crystalline phase. This phase accounts for almost 97.84 wt% of the total crystalline material, making it difficult to recognize minor reflections attributable to accessory crystals. Among these, a minor amount of aragonite has been detected, accounting for the remaining 2.16 wt%.

**FIGURE 10:**
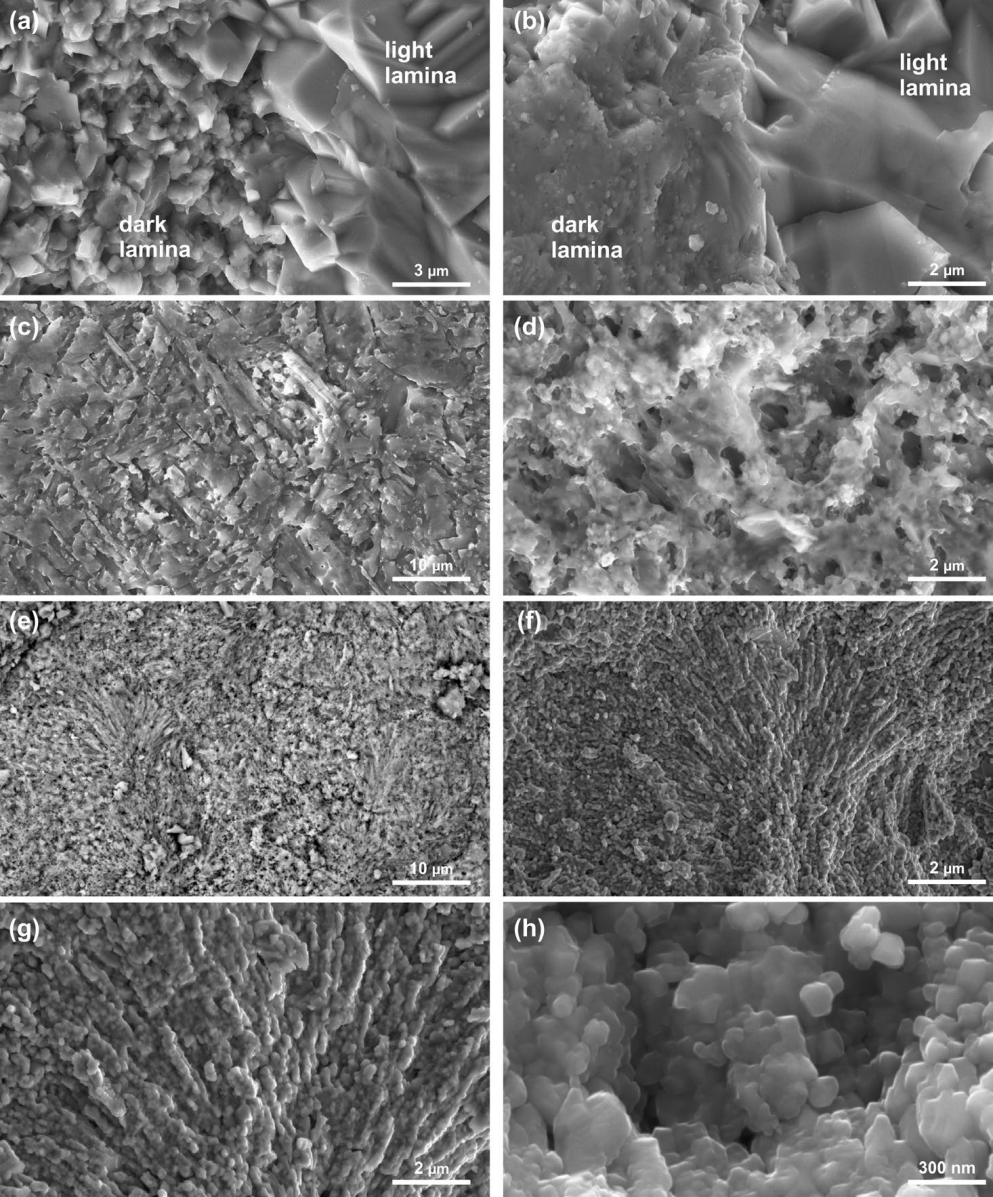
SEM images of dendrolite branches and thrombolite mesoclots: (a) Boundary between dark (microcrystalline) and light (macrocrystalline) dendroid laminae. (b) Recrystallized dendroid branch showing dark (anhedral crystals with fine corpuscles and amorphous materials) and light (euhedral crystals) laminae. (c, d) Weakly etched dendroid branch showing the remains of amorphous organic material and acicular crystals associated with the dark laminae. (e, f) Fan‐like nanotexture of acicular crystals forming the external portion of thrombolite spheroidal microstructures. (g, h) Amorphous organic material coating the thrombolite nanotexture.

Under SEM observation, due to recrystallization and resulting aggrading crystal size, the microlamination in the dendroids and the microfabrics of the surrounding micritic crusts, visible in optical microscopy, is not always well distinguishable. On unpolished fragments, the microlaminations appear as alternations of areas with different crystal size; microcrystalline areas tentatively correspond to the dark laminae, whereas macrocrystalline bands correspond to the lighter ones (Figure 10a). In the areas with stronger recrystallization, the two types of laminae can be distinguished by different crystal habits: anhedral crystals engulfing fine rounded nanometer particles and amorphous materials (tentatively interpreted as belonging to the dark laminae) pass to euhedral and well‐defined crystals (tentatively interpreted as belonging to the light laminae) (Figure 10b). Similarly, aphanitic and peloidal textures of the crusts show a microcrystalline arrangement, with macrocrystalline areas tentatively attributed to the sparite interclots.

The amorphous materials closely associated with the dark laminae are interpreted as organic matter remains. These are particularly evident on slightly etched thin sections of dark laminae, as well as in aphanitic/peloidal micrite samples. Weak acid application reveals the remains of acicular crystals engulfed in amorphous organic material (Figure 10c,d), testifying to a close relation of these fabrics with biotic activity.

EDS microanalyses reveal the presence of Ca (~97%wt), Sr (~1.5%wt), and Mg (~0.5%wt), which, together with the XRPD analyses and the remains of acicular crystals, suggest an original aragonitic composition for the dendroids fabric, subsequently calcitized during diagenesis. In contrast, a pure calcite composition was detected for microsparite from the lighter laminae and from the sparite filling of the microcavities.

#### 5.4.2 Thrombolites

XRPD analyses revealed calcitic composition for the thrombolite mesoclots. Calcite accounts for 98.99 wt%, and the remaining crystalline fraction is represented by hydroxyapatite (1.01 wt%). SEM observations show the external, acicular portion of the mesoclots micro‐spheroids to consist of very fine crystals (100 to 500 nm), organized in a fan‐like nanotexture which follows the upward growth direction of the thrombolite (Figure 10e,f). The nanocrystals do not show well‐defined boundaries; they are surrounded by amorphous material (Figure 10g,h) which during EDS analyses was burned by the electron beam, thus proving its carbonaceous nature.

The EDS microanalyses reveal the presence of only Ca (~98.8%wt) and Mg (~1.2%wt), suggesting a low‐magnesium calcite composition for the thrombolite mesoclots fabric. The pure calcite composition of the thrombolites is in stark contrast to that of the surrounding detritus, which contains variable amounts of Si, Al, K, and Fe, linked to the possible transport of clay minerals by diagenetic fluids within the porous sediment. The presence of apatite grains detected by SEM/EDS analyses may be related to very small vertebrate skeletal grains (probably fish remains) among the sediment. This agrees with the detection of hydroxyapatite by XRPD analyses.

## 6 Discussion

### 6.1 Nature of the DTB Dendrolites and Thrombolites

The results of the bio‐geochemical analyses reported above (UV epifluorescence, micro‐Raman, and SEM‐EDS) all indicate the presence of organic molecules, intimately associated with the microstructures of both dendrolites and thrombolites. This strongly suggests biotic mediation during the growth of the microstructures, putatively related to microbial associations. Hypotheses regarding the types of microbes involved may be derived from the analyses of the dendrolite and thrombolite microfabrics. In marine environments, the formation of microbial carbonates with distinctive microfabrics can be due to a complex combination of biotic and physicochemical factors, operating at both macro and microscale. Calcification of microbial communities to precipitate microbialite is largely a function of both alkalinity and availability of free calcium, which are combined in the saturation index (Riding 2000; Dupraz et al. 2009). Alkalinity may be influenced by physicochemical parameters of the macro‐environment and/or by microbial communities metabolically altering their immediate micro‐environment (Arp et al. 2001, 2003; Dupraz et al. 2009). This modification that leads to carbonate deposition can occur in both photosynthetic and heterotrophic microbes, via very different metabolic patterns (Merz‐Preiß and Riding 1999; Riding 2000; Arp et al. 2001; Dupraz et al. 2009). Production of EPS around bacterial cells also aids the nucleation and subsequent precipitation of calcium carbonate (Monty 1976; Chafetz 1986; Buczynski and Chafetz 1991; Reitner 1993; Kazmierczak et al. 1996; Folk and Chafetz 2000; Arp et al. 2001, 2003; Riding 2002; Riding & Tomás, Riding and Tomàs 2006).

Although the metabolic processes that induce microbialite precipitation can be complex and diverse, the resulting carbonate textures may be relatively few (Riding 2002, 2011). The most common microfabrics are laminated, peloidal, and aphanitic. This relative simplicity, together with the only rare preservation of the micro‐organisms involved and of other indicative features, greatly hampers reliable identification of the microbes and metabolic processes involved in microbial biomineralization.

In general terms, photosynthetic microbial activity may drive localized concentration gradients in water chemistry and mediate carbonate precipitation, forming thin wrinkled microlaminations (Davies 1970; Logan et al. 1974; Golubic 1985). Heterotrophic microbial communities, favored by adequate concentrations of organic matter, may instead produce microbial carbonates by ammonification, dissimilatory nitrate reduction, degradation of urea or uric acid, and sulfate reduction (Perry et al. 2007; Dupraz et al. 2009). These metabolic processes generate CO_3_^2−^, HCO^3−^, and ammonia (nitrogen metabolisms) or hydrogen sulfide (sulfate reduction) and cause a pH change that induces the formation of peloidal to clotted peloidal micrite textures. Furthermore, aphanitic micrite may result from the interaction of CaCO^3−^ supersaturated water with organic substrates, for example, decaying organic tissues such as biofilms or EPS.

In the Salento DTB, the fine‐laminated microfabric of the dendroids and of part of the associated micritic crusts may have been induced by autotrophic processes. Similar textures have been linked to coccoid and filamentous cyanobacteria (e.g., *Schizothrix*‐like taxa), potentially in association with putative EPS‐secreting diatoms (Davies 1970; Golubic 1985; Logan et al. 1974). These assemblages can thrive in shallow‐water settings, forming microbial films that, by superimposition, may gradually develop microlaminated dendroids. Microlaminated fabrics may also derive from heterotrophic bacterial activities, notably by sulfate‐reducing bacteria in anoxic pore‐water sediments or water columns (Visscher et al. 2000; Dupraz et al. 2009), or by chemotrophic bacteria and archaea (Bailey et al. 2009). However, the overall sedimentary context and the comparison of the microlaminated texture with other cyanobacterial‐mediated microbialites (Davies 1970; Golubic 1985; Logan et al. 1974) suggest that heterotrophic microbial processes in this mineralization are unlikely. The possible presence of heterotrophic bacteria, on the other hand, seems to be linked to the immediate surroundings of the dendroids. The abundant cavities within the dendrolite framework possibly allowed the accumulation of organic matter, deriving from microbial metabolic activities and/or EPS production. This, in turn, may have stimulated the development of heterotrophic microbial communities with the formation of clotted peloidal and aphanitic micrite crusts among the dendroids. The particular arrangement of these crusts, enveloping and coating the dendroid branches, further confirms their microbial nature. Most probably, this micro‐environment was characterized by restricted water circulation and low oxygen content. Similar redox conditions, associated with the deposition of heterotrophic microbial micrite, have been described by Tosti et al. (2014) for the skeletal framework in Carnian patch reefs of Alpe di Specie (Italian Dolomites) and by Heindel et al. (2010) for Pleistocene–Holocene reefs (Tahiti). Lipid biomarkers indicating a bacterial community dominated by sulfate‐reducing bacteria that degraded organic matter may be linked to peloidal to aphanitic autochthonous micrite precipitation in cryptic bioconstructions of recent (Guido et al. 2012, 2013, 2016) and Pleistocene (Guido et al. 2017) submarine caves. At the same time, part of the organic matter may have influenced organomineralization processes to produce aphanitic micrite (Guido et al. 2022).

The nature of the DTB thrombolite microbial association is particularly difficult to infer because—so far we are aware—their particular microfabric seems to be extremely rare. In general terms, thrombolites are products of complex and varied associations dominated by photosynthetic prokaryotes (i.e., cyanobacteria and purple sulphur bacteria), eukaryotic microalgae (i.e., diatoms) and to a lesser extent, chemoautotrophic and chemoheterotrophic microbes (Moore 1991). The upward growth direction of DTB thrombolite mesoclots appears phototactic, but the apparent absence of trapped sediment and micritic and peloidal coatings could reflect a reduced presence or complete absence of microalgal EPS. The possible relation with cyanobacterial activity seems supported by one of the few other examples of fossil thrombolites showing a somewhat comparable microfabric. Arborescent thrombolites from the middle‐late Cambrian of central Australia (Kennard 1994) are characterized by a microstructure of poorly defined spherulites with a radial fibrous texture, interpreted as the product of calcification of coccoid cyanobacteria.

Mineralogical characterization points to an originally aragonite nature for the dendrolite, subsequently calcitized during diagenesis, whereas thrombolite could preserve original calcite. These different mineral phases among the two microbialite frameworks, as well as the dual nature of the thrombolite mesoclots spheroidal microstructures, with a nucleus of sparry calcite surrounded by a generation of acicular crystals, are puzzling. Further detailed micromorphological and geochemical characterization is needed to confidently interpret them in terms of specific microbial mediation, particular environmental parameters, or diagenetic processes. Actually, microbialites form in a broad range of marine and continental settings (e.g., hot springs, freshwater rivers and lakes, hypersaline, and/or alkaline lakes). The wide variability of physicochemical parameters, such as alkalinity, salinity, (Mg/Ca)_aq_ ratio, and dissolved Mg^2+^ and H_4_SiO_4_ concentrations (Müller et al. 2022) controls microbialite morphology, fabric, and mineralogical and chemical composition. Consequently, defining specific microbial processes and understanding how these processes influence the thermodynamic equilibrium between aragonite and calcite could help clarify the distribution of dendrolites vs. thrombolites in the studied build‐up.

### 6.2 DTB Palaeoenvironmental Setting and Depositional Model

Vescogni et al. (2022) report normal marine salinity and shallow‐water characterized by high‐energy conditions for the basal cycle of the SP‐TCC (CLS, SCB, MCC, and OG facies, Figure 3c, Table 1). This is particularly evident for the MCC facies, characterized by coarse grainstone and the presence of ooids, coralline red algae, echinoderms, and dasycladacean algae. MCC deposits completely surround and cover the DTB (Figure 3c). Combined with the lack of sharp boundaries between the two facies, this suggests that the MCC and DTB developed under similar environmental conditions. However, the sediment among the dendrolites and thrombolites shows slightly different features in comparison to the MCC: the former is actually a finer packstone–grainstone, which lacks the typical markers of normal marine salinity, such as green and coralline red algae and echinoderms. The MDT depositional setting could thus have been characterized by lower hydrodynamic conditions and a salinity level that deviated from normal values. In addition, the lack of abrasion, bioerosion, and encrustations on the small branches possibly indicate fast burial of the dendrolitic structures under a relatively high sedimentation rate.

Examples of microbial carbonates made by microlaminated dendrolites seem to be rare in the geological record and mainly related to centimeter‐thick encrustations. However, the related paleoenvironmental reconstructions suggest depositional settings quite similar to that of the Salento build‐up. Late Cambrian/Early Ordovician dendrolites from Texas are few centimeters tall, thick‐branched dendroids that grew in shallow cavities within decimetric rimmed columns, the latter with an external border made of microlaminated stromatolite (Lee and Riding 2023). These structures formed in a shallow‐water, high‐energy, normal marine environment where the dendroids could have been forced to grow in response to sediment accumulation that occurred preferentially in the hollow, inner part of the columns (Lee and Riding 2023). Upper Triassic microlaminated dendrolites described from southwest Britain occur within a relatively thin horizon of microbial carbonates, 20 to 250 cm thick, but with an overall lateral extent of several tens of kilometers (Ibarra et al. 2014). These finely digitate dendroids, a few mm up to 5 cm tall, are distributed along sub‐horizontal intervals that alternate with stromatolitic layers. They have been interpreted as the product of microbial activity in a shallow‐water setting during the period of high *p*CO_2_ and relatively warmer conditions associated with the mass extinction that characterized the end‐Triassic (Ibarra et al. 2014).

**FIGURE 11:**
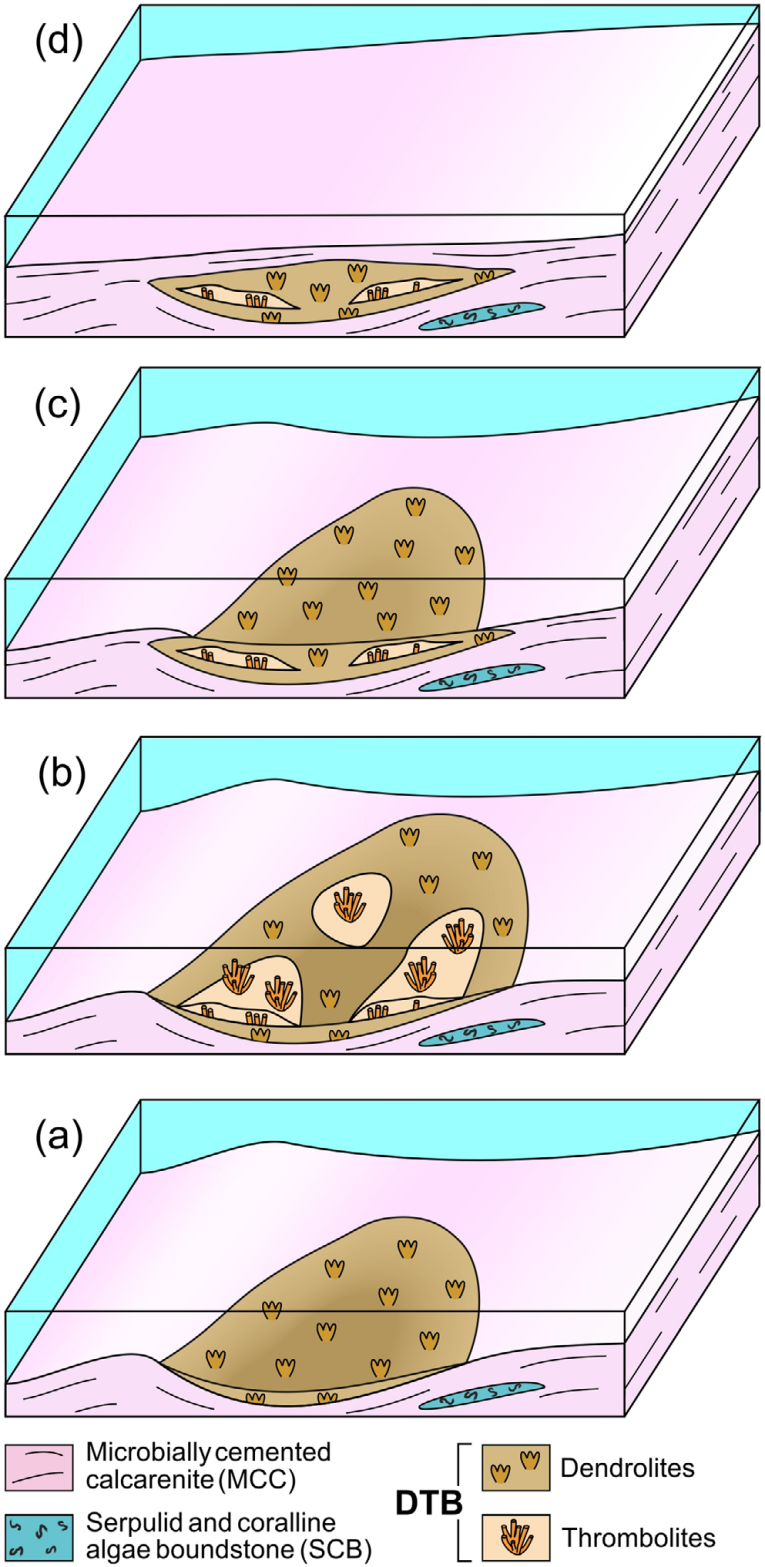
Reconstruction of DTB depositional phases: (a) Dendrolite colonization commences in a shallow‐water environment with relatively moderate water movement and high salinity; possibly a small lagoon confined seaward by MCC shoals. (b) Thrombolite patches colonize the dendrolite. (c) Renewed dendrolite growth together with partial burial of the DTB by adjacent MCC sediments. (d) MCC sediments completely cover the DTB, terminating microbial colonization.

The development of the Salento DTB could thus have started within a shallow, confined portion of the sea floor, possibly a small lagoon protected from the open sea by sand shoals of MCC facies. This environment is likely to have experienced high evaporation rates, with high salinity coupled with euphotic and moderate hydrodynamic conditions. This setting may have supported a microbial assemblage dominated by putative *Schizothrix*‐like cyanobacteria and EPS‐secreting diatoms, leading to the first growth stage of the DTB dendrolites (Figure 11a). A similar model was established by Monty (1976) for microbialite sediments deposited along the eastern Andros Island. The initial dendrolite growth phase would then be followed by the development of thrombolite, represented at first by small, scattered growth forms (thin‐branched thrombolites), followed by larger, more extended patches (thick‐branched thrombolites).

The turnover between dendrolites and thrombolites is not easily explained by our data. The fundamental differences between their mineral composition and microstructure suggest a putative radical change in the microbial association; this may have been related to variations in salinity and/or in sediment supply (Browne et al. 2000). For example, the temporary onset of a humid phase could have significantly reduced salinity, possibly altering the environmental saturation index and triggering a turnover in microbial colonization. Under these conditions, the original microbial communities may have been replaced by bacteria adapted to freshwater environments, such as the *Scytonema* assemblage described by Monty (1976), ultimately leading to the development of the thrombolite horizon (Figure 11b). In addition, the anastomose growth form of the thrombolites possibly suggests an enhanced sedimentation rate, as microbial associations often develop upward branching morphologies in reaction to sediment accumulation (see e.g., Mackey et al. 2015; Lee and Riding 2023). At the end of this phase, the environment could return to the initial conditions, and the recovery of the dendrolitic microbial assemblage. The transgressive trend characterizing the sequence containing the DTB (see Vescogni et al. 2022) could have led to a progressive increase in relative sea level and partial burial of the dendrolites by MCC sediments (Figure 11c). This trend would have finally led to complete coverage of the MDT and definitive cessation of DTB development (Figure 11d).

## 7 Conclusions

The composite Messinian dendrolite/thrombolite build‐up cropping out in the Salento Peninsula (Italy) is unique in the panorama of microbial sedimentation. This structure is part of a shallow‐water carbonate succession characterized by different microbial facies so far never recorded in the Late Miocene of the Mediterranean.

Bio‐geochemical analyses (UV epifluorescence, micro‐Raman spectroscopy, and SEM‐EDS) strongly support a biotic origin for both dendrolites and thrombolites. Although direct evidence of specific micro‐organisms is lacking, the microfabrics and associated sedimentary context are consistent with the potential involvement of cyanobacterial communities in their development, possibly alongside a subordinate contribution from heterotrophic microbial consortia. However, this interpretation remains speculative, and future investigations aimed at identifying potential lipid biomarkers could better constrain the microbial metabolisms and bio‐geochemical processes involved in mineralization.

Microlaminated dendroids show an original aragonitic composition. Previously known mainly from the Cambrian and Triassic, until now they have not been generally associated with the construction of large build‐ups.

Thrombolites display an original calcitic composition. The mesoclots microfabric shows a particular texture made of spheroidal aggregates with an internal nucleus of sparry calcite surrounded by a rim of acicular crystals. The precise understanding of the genesis of this texture is still unclear and needs further bio‐geochemical analyses.

The Salento microbial build‐up developed in a small, shallow‐water lagoon, with moderate to high hydrodynamic conditions and salinity values influenced by alternatively high evaporation rates and possible freshwater input. The branched/anastomose growth pattern of dendrolites and thrombolites could be related to relatively high sedimentation rates.

The abrupt change from the small dendroids of the dendrolites to the larger thrombolite mesoclots, coupled with major changes in their microfabrics and mineral composition, could be related to a radical change in the microbial assemblages, possibly forced by environmental perturbations in salinity and/or in sediment supply.

## Conflicts of Interest

The authors declare no conflicts of interest.

## Data Availability

The data that support the findings of this study are available from the corresponding author upon reasonable request.
